# The human H5N1 influenza A virus polymerase complex is active *in vitro* over a broad range of temperatures, in contrast to the WSN complex, and this property can be attributed to the PB2 subunit

**DOI:** 10.1099/vir.0.2008/006254-0

**Published:** 2008-12

**Authors:** Birgit G. Bradel-Tretheway, Z. Kelley, Shikha Chakraborty-Sett, Toru Takimoto, Baek Kim, Stephen Dewhurst

**Affiliations:** Department of Microbiology and Immunology, University of Rochester Medical Center, Rochester, NY 14642, USA

## Abstract

Influenza A virus (IAV) replicates in the upper respiratory tract of humans at 33 °C and in the intestinal tract of birds at close to 41 °C. The viral RNA polymerase complex comprises three subunits (PA, PB1 and PB2) and plays an important role in host adaptation. We therefore developed an *in vitro* system to examine the temperature sensitivity of IAV RNA polymerase complexes from different origins. Complexes were prepared from human lung epithelial cells (A549) using a novel adenoviral expression system. Affinity-purified complexes were generated that contained either all three subunits (PA/PB1/PB2) from the A/Viet/1203/04 H5N1 virus (H/H/H) or the A/WSN/33 H1N1 strain (W/W/W). We also prepared chimeric complexes in which the PB2 subunit was exchanged (H/H/W, W/W/H) or substituted with an avian PB2 from the A/chicken/Nanchang/3-120/01 H3N2 strain (W/W/N). All complexes were functional in transcription, cap-binding and endonucleolytic activity. Complexes containing the H5N1 or Nanchang PB2 protein retained transcriptional activity over a broad temperature range (30–42 °C). In contrast, complexes containing the WSN PB2 protein lost activity at elevated temperatures (39 °C or higher). The E627K mutation in the avian PB2 was not required for this effect. Finally, the avian PB2 subunit was shown to confer enhanced stability to the WSN 3P complex. These results show that PB2 plays an important role in regulating the temperature optimum for IAV RNA polymerase activity, possibly due to effects on the functional stability of the 3P complex.

## INTRODUCTION

Among the viral factors that contribute to virulence and host range of influenza A viruses (IAVs) are the viral polymerase proteins (Almond, 1977; Gabriel *et al.*, 2005, 2007; Hatta *et al.*, 2001; Murakami *et al.*, 1988). The IAV polymerase is a heterotrimeric protein (3P) complex comprised of PB1, PB2 and PA. PB1 contains conserved sequence elements found in other RNA-dependent RNA polymerases (Deng *et al.*, 2005; Fodor & Brownlee, 2002; Lamb & Krug, 2001), and binds directly to the viral (vRNA) and complementary RNA (cRNA) promoter sequences. PA is needed for transcription and replication (Fodor *et al.*, 2002; Hara *et al.*, 2006; Perales *et al.*, 2000; Regan *et al.*, 2006), and for efficient nuclear accumulation of PB1 (Fodor & Smith, 2004; Sanz-Ezquerro *et al.*, 1995). The third subunit, PB2, localizes to the nucleus and to mitochondria (Carr *et al.*, 2006). It binds to cap structures of host mRNAs which are then cleaved by the endonucleolytic activity of PB1 (Guilligay *et al.*, 2008; Li *et al.*, 2001; Plotch *et al.*, 1981). PB2 also plays a major role in host adaptation (Finkelstein *et al.*, 2007; Gabriel *et al.*, 2008; Hatta *et al.*, 2001; Labadie *et al.*, 2007; Li *et al.*, 2005; Naffakh *et al.*, 2000; Salomon *et al.*, 2006). One factor that contributes to host adaptation is the temperature optimum for virus replication. IAV replicates in the upper respiratory tract of humans at 33 °C and in the intestinal tract of birds close to 41 °C. In cell culture, human IAV can replicate at temperatures ranging from 33 °C to 37 °C, but not at elevated temperatures such as 42 °C. In contrast, most avian IAVs exhibit cold sensitivity with reduced replication at 33 °C, but efficient replication at 37 °C or 42 °C (Massin *et al.*, 2001; Murakami *et al.*, 1988; Murphy *et al.*, 1982a, b).

It has been shown that amino acid residue 627 in PB2 is one of the main determinants for cold sensitivity of the avian IAV and for viral pathogenicity in mammalian hosts (Hatta *et al.*, 2007; Massin *et al.*, 2001; Murakami *et al.*, 1988; Torreira *et al.*, 2007): the presence of lysine at position 627 (K^627^), instead of the glutamic acid (E^627^) that is found in avian viruses, is crucial for high virulence in mice (Hatta *et al.*, 2001). This amino acid does not determine the cell tropism of the virus (Shinya *et al.*, 2004), but instead enhances viral growth in mice and probably in humans (Hatta *et al.*, 2007; Salomon *et al.*, 2006). The mechanism underlying this observation still needs to be elucidated.

Most studies on temperature sensitivity of IAVs have been performed at the level of viral transcription-replication. We were interested in how different temperatures directly affect viral RNA polymerase activity at the transcriptional level. To do this, we elected to use two well-characterized IAVs: (i) the mouse-adapted A/WSN/33 H1N1 virus (‘WSN’) and (ii) the A/Viet/1203/04 H5N1 virus (‘VN1203’). WSN has been described to replicate with about tenfold lower efficiency at 39.5 °C compared with 34 °C, as measured by plaque assay (Kawaguchi *et al.*, 2005). In contrast, the VN1203 virus has been shown to replicate efficiently at both low (34 °C) and high temperatures (41 °C) (Hatta *et al.*, 2007).

We purified 3P complexes (PA/PB1/PB2) from the VN1203 (H/H/H) and WSN (W/W/W) strains and compared the effect of temperature on cap-independent and cap-dependent transcription. In addition, we exchanged the PB2 subunits between these two complexes (H/H/W, W/W/H) or substituted the PB2 subunit with an avian subunit from the A/chicken/Nanchang/3-120/01 strain (H3N2) (W/W/N). We developed an adenoviral expression system which is attractive, because recombinant adenoviruses can enter a broad range of cells, including IAV-susceptible human lung epithelial cells (A549). As a result, it is possible to prepare authentic IAV polymerase complexes that contain associated host cell factors that participate in complex formation and/or polymerase function. Using this approach, we show here that PB2 directly affects the temperature optimum for IAV transcription, and that incorporation of an H5N1 or avian PB2 subunit into the IAV 3P complex results in greatly increased polymerase activity at elevated temperatures characteristic of birds. We also show that this effect of the H5N1 and avian PB2 subunits does not depend on the presence of a glutamic acid residue at position 627 of PB2. Finally, our results suggest that PB2 may exert its effect on the temperature optimum of the IAV RNA polymerase by influencing the functional stability of this trimeric protein complex.

## METHODS

### Influenza virus strains, vectors and cells.

Each polymerase subunit of the WSN (H1N1) strain (A/WSN/33) was subcloned from pcAGGS-PA/PB1/PB2_WSN_ (gift of Dr Y. Kawaoka, University of Wisconsin-Madison, WI, USA). The PB2 gene was amplified by reverse-transcription PCR from the Nanchang (H3N2) strain (A/chicken/Nanchang/3-120/01), obtained from Dr R. Webster, St Jude Children's Research Hospital, Memphis, TN, USA (Liu *et al.*, 2003). The E627K mutation was introduced by site-directed mutagenesis (Quick-Change mutagenesis kit, Stratagene). The polymerase genes of the VN1203 (H5N1) strain (A/Vietnam/1203/04) were assembled from synthetic oligonucleotides by GENEART (GenBank accession nos EF467808, AY818132.1, AY651719.1).

PA from the WSN and the VN1203 strain were tagged at the C terminus with a tandem-affinity purification (TAP)-tag. The TAP-tag was constructed by overlapping PCR-mutagenesis, resulting in a C-terminal TAP where the thrombin cleavage site was followed by a 6×His tag, a tobacco etch virus (TEV) cleavage site and an IgG-binding domain (protein A domain). Briefly, the ZZ domain of BG1766 (Malkowski *et al.*, 2007) was amplified by overlapping PCR introducing a TEV cleavage site. The amplified PCR product was ligated at the 5′-terminal *Eco*RV site to a dsDNA fragment (TCTAGACTGGTGCCGCGCGGCAGCCATCATCATCACCATCATGATATC) containing a thrombin site and a 6×His tag. WSN or VN1203 PA were fused at their C terminus to the TAP-tag.

Human embryonic kidney (HEK293A or QBI-HEK 293CymR) or human lung endothelial cells (A549) were grown at 37 °C and maintained in DMEM supplemented with 10 % fetal bovine serum, 50 IU penicillin ml^−1^, 50 μg streptomycin ml^−1^ and 2 mM glutamine.

### Adenovirus production.

Recombinant adenoviruses expressing either LacZ or PB2 from the Nanchang strain (N or N_E627K_) or PA-TAP, PB1 or PB2 from the WSN or the VN1203 strain were created from pShCMV by homologous recombination into AdEasy and propagation in HEK293A cells (AdEasy system, Stratagene). Only the adenovirus expressing PB2 from the VN1203 strain was constructed differently, by cloning PB2 into the pAdenoVator-CMV(CuO) plasmid followed by recombination into AdEasy and propagation in QBI-HEK 293CymR cells (Qbiogene). The viruses were purified by CsCl density-gradient centrifugation and dialysis. Viral titres were determined by quantitative real-time PCR (Heim *et al.*, 2003).

### Polymerase purification.

A549 cells (6×10^6^ in a T-225 flask) were transduced the following day with each of the three adenoviruses (rAd5-PB1, -PB2, -PA-TAP) at an m.o.i. of 1000 (80 % transduction efficiency); for adenoviruses expressing the codon-optimized genes PA, PB1 and PB2 from the VN1203 (H5N1) strain an m.o.i. of about 100 was used.

Nuclear and cytoplasmic extracts were prepared 48 h post-infection (Nuclear Extract kit, Active Motif) and purified using the TAP method (described below).

Lysates from whole cells were prepared 48 h post-infection in 3 ml ice-cold 1× RIPA buffer (Upstate) supplemented with 1 mM NaF, 1 mM PMSF, 1 mM NaVO_3_, 1 μl Protease Inhibitor Cocktail (P8340, Sigma).

Poly-Prep Chromatography Columns (0.8×4 cm, Bio-Rad) were loaded with 300 μl IgG Sepharose 6 fast flow bead suspension (Amersham) and washed with IPP-150 buffer (Puig *et al.*, 2001). Pre-cleared cell lysate (3 ml, corresponding to cells from one 225 cm^2^ flask) was mixed with 7 ml IPP-150 buffer and rotated for 2–4 h at 4 °C in the presence of IgG. The columns were washed with IPP-150 then with TEV-cleavage buffer (IPP-150 supplemented with 1 mM DTT and 0.5 mM EDTA). The beads were transferred to Micro Bio-Spin Chromatography columns (Bio-Rad) and incubated at 4 °C overnight in 300 μl TEV buffer containing TEV-enzyme (30 U). The eluate was collected by low speed centrifugation and dialysed (50 mM Tris-Cl (pH 7.5), 1 mM EDTA, 200 mM NaCl, 10 % glycerol, 1 mM DTT) using a 20 000 MWCO Dialysis Cartridge (Pierce).

### Silver staining and immunoblotting.

Proteins were heat-denatured and separated by 7.5 % SDS-PAGE prior to immunoblotting. For silver staining, gels were treated according to the manufacturer's protocol (SilverXpress Silver Staining kit, Invitrogen).

Antibodies used were (Santa Cruz): anti-*α*-tubulin (mouse, TU-02), anti-PB1 (vK-20), anti-PB2 (vN-19), anti-actin (H-196) and anti-goat IgG HRP (Sc-2033). ECL anti-rabbit IgG HRP (NA934V) and ECL anti-mouse IgG HRP (NA931V) were purchased from Amersham and anti-Penta-His (mouse) from Qiagen.

### ApG- and globin-primed transcription assay.

The ApG-primed transcription assay was performed basically as described elsewhere (Fodor *et al.*, 2002). The amount of purified 3P complex used in transcription assays was normalized either in terms of PB2 content (as measured by immunoblotting) or in terms of transcriptional activity at 30 °C. IgG-purified, normalized samples were mixed with transcription buffer (25 mM Tris-Cl, pH 7.5, 100 mM KCl, 5 mM MgCl_2_, 0.1 mM EDTA, 2 mM DTT, 0.25 Nonidet P-40, 12.5 % glycerol) supplemented with 0.25 U RNasin μl^−1^, 1 mM each ATP/ UTP/CTP, 0.1 μM GTP, 0.15 μM [*α*-^32^P]GTP (3000 Ci mmol^−1^), 1.6 μM each 5′- (5′-AGUAGAAACAAGGCC-3′) and 3′-end vRNA (5′-GGCCUGCUUUUGCU-3′) and 0.3 mM ApG (Biosynthesis). The reaction volume (5 μl) contained 2 μl normalized protein sample. Transcription was allowed to occur for 1 h at 30, 34, 37, 39 or 42 °C and the reaction was terminated by the addition of 4 μl Stop-buffer (10 mM EDTA pH 8.0, 90 % formamide). Transcription products were separated by 18 % PAGE in 7 M urea and detected by autoradiography using a PhosphorImager (Molecular Dynamics). Densitometry was performed using OptiQuant (Version 3.10, Packard Instrument).

The globin-primed (cap-dependent) transcription assay was performed similarly. Instead of ApG rabbit-globin mRNA (Sigma) was used as a cap-donor (10 ng per 5 μl reaction volume) and the incubation time was 90 min (Hara *et al.*, 2006).

### Thermo-stability assay.

Equivalent amounts of polymerase in 2 μl (as determined by polymerase activity at 30 °C) were pre-incubated at 30 °C for 5, 10, 15, 20, 25, 30, 60 and 120 min. After pre-incubation 3 μl ApG-containing transcription buffer, supplemented as described above, was added to the sample and transcription was allowed to occur at 30 °C for 1 h.

## RESULTS

### Adenoviral expression of the IAV polymerase in human lung epithelial cells (A549)

A549 cells were transduced with either a LacZ-expressing control adenovirus, a PA_WSN_-TAP-expressing virus or a combination of three viruses expressing PA_WSN_-TAP, PB1_WSN_ and PB2_WSN_ (W/W/W). To test whether the adenovirus-mediated expression system might alter the normal distribution of the IAV 3P complex, we analysed the cellular distribution of PA in the absence or presence of PB1 and PB2. To do this, nuclear and cytoplasmic extracts were prepared from vector-transduced A549 cells, separated by SDS-PAGE and blotted with antibodies directed against PA, PB1 and PB2.

**Fig. 1.**
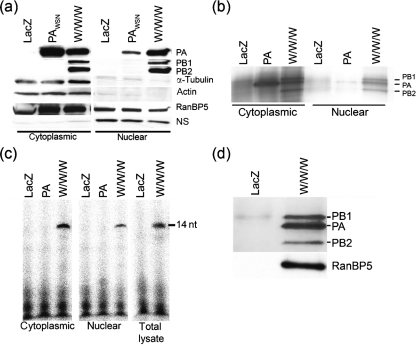
Subcellular distribution and activity of purified, adenovirus-expressed WSN polymerase. (a) A549 cells were transduced with rAd5-PA_WSN_-TAP alone or with a combination of rAd5-PA_WSN_-TAP, -PB1_WSN_ and -PB2_WSN_ (W/W/W). Nuclear and cytoplasmic extracts were analysed by immunoblotting using PA-, PB1- or PB2-specific antibodies. The effectiveness of the subcellular fractionation method was confirmed by probing against the cytoplasmic proteins, actin and *β*-tubulin as well as against RanBP5 (which is found both in the cytoplasm and nucleus). ns, non-specific protein band detected when probing against PB1/2. Cells transduced with an adenovirus vector expressing LacZ served as a negative control. Twofold more cell equivalents were loaded for the nuclear extracts than the cytoplasmic extracts. (b) IgG-purified nuclear and cytoplasmic extracts were analysed by silver staining prior to (c) comparison with total cell lysate in an ApG-primed transcription assay (1 h at 30 °C). Extracts from equivalent numbers of transduced A549 cells were used. (d) IgG-purified extracts from total cellular lysates were analysed by silver staining (upper panel) and immunoblotting against RanBP5 (lower panel). The positions of PA, PB1 and PB2 are shown on the right.

To confirm the effectiveness of our subcellular fractionation method, we also examined the presence of *α*-tubulin and actin (both cytoplasmic proteins), and of RanBP5 (which is found predominantly in the cytoplasm, but also in the nucleus) (Deane *et al.*, 1997). As shown in Fig. 1(a), nuclear fractions were lacking in both *α*-tubulin and actin, but contained RanBP5. Fig. 1(a) also shows that PA, when expressed alone, mainly accumulated in the cytoplasm with less than 10 % in the nucleus; in contrast, when co-expressed with PB1 and PB2 (W/W/W), PA was readily detected in the nucleus (about 30 %), as expected (Fodor & Smith, 2004; Nieto *et al.*, 1992). Thus, the adenovirus expression system had no detectable effect on the expected subcellular localization of the IAV 3P complex and its individual component proteins.

### Transcriptional activity of the WSN 3P complex purified from A549 cells

The polymerase complex was purified from whole-cell lysates, nuclear and cytoplasmic extracts by binding PA-TAP via its protein A domain to IgG-beads. All proteins that interact with PA, including PB1, PB2 and host factors binding to the 3P complex, were expected to be pulled down by this approach. The complex was released from the beads by cleavage with TEV-enzyme and the purity of the complex was analysed by silver staining (Fig. 1b, d).

We next tested the transcriptional activity of the purified polymerase complexes using an ApG-primed transcription assay in which the 3′- and 5′-vRNA and the dinucleotide ApG were provided. In this assay, the 3P complex initiates extension of the bound dinucleotide; the fully extended product of this reaction is a 14 nt RNA fragment. No extension products were detected when the ApG-primed transcriptional assay was performed with IgG-purified extracts from cells expressing LacZ or PA alone. Only the fully assembled trimeric polymerase complex (W/W/W) generated an extension product (Fig. 1c). We did not observe any difference in transcriptional activity of purified polymerase complexes from total cell lysates, or from either nuclear or cytoplasmic extracts (Fig. 1c); the apparently lower transcriptional activity of the nuclear polymerase complex can be attributed simply to the lower amount (∼30 % less) of assembled 3P complex present in the nucleus (Fig. 1b). For further biochemical studies, 3P complexes were isolated in larger quantities from total cell lysates and analysed by silver staining (Fig. 1d, top panel). The most abundant protein in the purified complex was PA, which was precipitated as a monomer, or complexed with either PB1 (PA/PB1) or PB1 and PB2 (PA/PB1/PB2). The second most abundant protein was PB1, which was pulled down as a dimeric (PA/PB1) or trimeric (PA/PB1/PB2) complex. The presence of dimeric (PA/PB1) complex can be inferred by the presence of the host-factor RanBP5 (Fig. 1d, bottom panel; Supplementary Fig. S1, available in JGV Online) that is known to interact with PB1 or PB1/PA (Deng *et al.*, 2006). PB2 was the least abundant protein since it was only precipitated in the context of the intact 3P complex (PA/PB1/PB2); therefore in our future experiments, PB2 content was used to normalize overall levels of the intact 3P complex (unless otherwise indicated). For all following experiments 3P complexes purified from total cellular lysates were utilized.

### The 3P complex from the A/Viet/1203/04 (VN1203) strain is highly active, and both the WSN and VN1203 complexes are competent for cap-independent and cap-dependent transcription

**Fig. 2.**
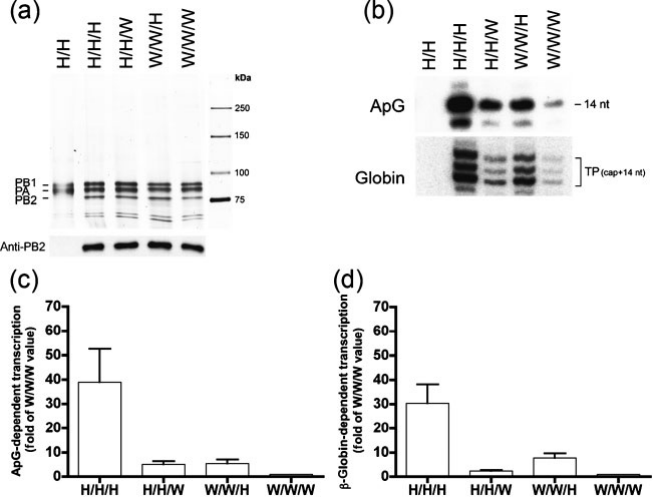
The H5N1 3P complex is more active than the WSN 3P complex in both cap-independent and cap-dependent transcription. (a) Purified 3P complexes from the VN1203 (H/H/H) and WSN (W/W/W) viruses, along with chimeric (H/H/W and W/W/H) and dimeric complexes (H/H; negative control), were detected by silver staining and immunoblotting against PB2 (top and bottom panel, respectively). PB2-normalized complexes were used in functional assays (30 °C). (b) The complexes were analysed for cap-independent (ApG) or cap-dependent transcription (top and bottom panel, respectively). The transcription products in the ApG-primed assay (14 nt long transcript) and in the globin-primed assay (triplet band; capped primer +14 nt) were detected by autoradiography. (c, d) Quantitative data for the ApG- and the cap-RNA-primed assays are shown. The data were normalized in terms of fold activity over the activity of the W/W/W complex. The results shown represent mean transcriptional activity data from at least three independent assays. Bars, sem.

We purified 3P complexes (PA/PB1/PB2) from the VN1203 (H5N1) (H/H/H) and the A/WSN/33 (H1N1) (W/W/W) strains as well as complexes in which the PB2 subunits were exchanged (H/H/W, W/W/H) or omitted (H/H). The purified complexes were analysed by silver staining and immunoblotting against PB2 (Fig. 2a top and bottom panel, respectively). All complexes, except the negative control (H/H), were able to initiate transcription at 30 °C in a cap-independent fashion by utilizing ApG as a primer (Fig. 2b, top panel). A fully extended primer that yielded a 14 nt transcript was detected for all complexes. An additional band above the 14 nt extension product is likely due to some terminal transferase ability of the IAV polymerase.

Influenza viruses use a cap-snatching mechanism to initiate transcription, which involves recognition and binding of the cap structure of host mRNAs by PB2, followed by cleavage of the capped mRNA at nucleotide 10–14 by the endonucleolytic activity of PB1 (Guilligay *et al.*, 2008; Li *et al.*, 2001; Plotch *et al.*, 1981). These short RNAs are then utilized by the polymerase as primers to initiate transcription. We expected that cleavage of the provided cap-donor by the 3P complex would result in the generation of short RNAs able to bind to the 3′-vRNA template (14 nt). The resulting transcription product (cap primer +14 nt) was detected as a triplet band in our assays (Fig. 2b, bottom panel), which could be due to multiple cleavage sites in the primer or to residual nuclease activity (Fodor *et al.*, 2002). All purified complexes were able to utilize rabbit globin mRNA to prime transcription. Thus, all of the complexes, except the negative control (H/H), were competent for cap-binding and endonucleolytic activity. Only a fully assembled, trimeric complex is transcriptionally active (Deng *et al.*, 2005; Perales & Ortin, 1997).

Relative levels of transcriptional activity for the various complexes were measured by normalizing to the levels of PB2 (Fig. 2a, bottom panel). This analysis revealed that the VN1203 3P complex was 40-fold more active in the ApG-primed (Fig. 2c) and about 30-fold more active in the cap-primed transcription assay (Fig. 2d) than the WSN 3P complex, and that the PB2 subunit contributes to this enhanced activity. These findings are consistent with those reported by Taubenberger, who noted that IAV polymerase complexes containing avian subunits have elevated *in vitro* activity compared with those containing human IAV proteins (Taubenberger *et al.*, 2005). The biological significance and mechanistic underpinnings of this observation will require further investigation.

### The VN1203 polymerase is active at elevated temperatures, and the PB2 subunit is sufficient to confer broad temperature tolerance on the WSN polymerase

**Fig. 3.**
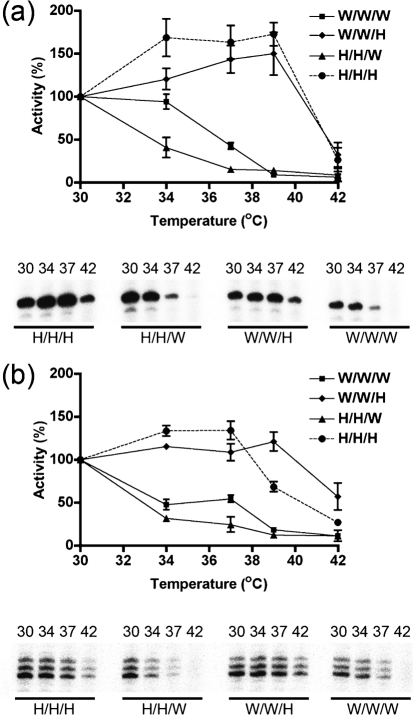
The human H5N1 3P complex is active at high temperatures and the H5N1 PB2 subunit confers this same phenotype on the WSN 3P complex. Equivalent amounts of polymerase complex (as determined by polymerase activity at 30 °C) were tested for transcriptional activity at different temperatures (30, 34, 37, 39, 42 °C). (a, b) Representative autoradiograms and quantitative data (top and bottom panel, respectively) for the ApG- (a) and the cap-RNA-primed (b) assays are shown. The data were normalized in terms of percentage of the amount of fully extended product at 30 °C (as quantified by Phosphorimager analysis); the results shown represent mean transcriptional activity data from at least four independent assays. Bars, sem.

The purified polymerase complexes were subjected to the ApG- (Fig. 3a) or globin-primed (Fig. 3b) transcription assay. Reactions were run at 30 °C and at a range of temperatures selected on the basis of their physiological relevance to influenza virus replication (34, 37, 39 and 42 °C). In these experiments, the amount of input 3P complex was standardized by using an equivalent amount of functionally active complex, as determined on the basis of transcriptional activity at 30 °C. The WSN complex (W/W/W) extended the ApG primer best at temperatures ranging from 30 to 34 °C; activity was reduced by more than twofold at 37 °C and more than tenfold at elevated temperatures (39–42 °C) (Fig. 3a). In contrast, the VN1203 complex (H/H/H) extended the primer best at 34–39 °C, and retained 25 % of its activity at elevated temperature (42 °C). Chimeric complexes in which the PB2 subunit was exchanged (H/H/W, W/W/H) exhibited a temperature sensitivity profile similar to that of the WT complexes from which the PB2 subunit was derived – i.e. the H/H/W complex was minimally active at elevated temperatures, while the W/W/H complex retained a high level of functionality at 39–42 °C (Fig. 3a). In contrast, the presence of excess PA/PB1 heterodimers in the WSN complexes (W/W/W) had no effect on the thermotolerance of the polymerase (Supplementary Fig. S1).

Results from the cap-primed transcription assay (Fig. 3b) were essentially equivalent to those obtained from the ApG-primed assay. The W/W/W complex extended the capped mRNA best at 30 °C, and its activity was reduced to 50 % at 34–37 °C and to less than 20 % at 39–42 °C. In contrast, the H/H/H complex extended the capped primer best at 30–39 °C, and retained significant activity at 42 °C. In this assay, the temperature sensitivity profile of the activity of the chimeric complexes was again determined by the PB2 subunit.

Overall, results obtained with the cap-independent and cap-dependent transcription assays were very similar. One minor difference was that, in the ApG-primed transcription assay, we observed a somewhat more pronounced difference in temperature sensitivity between complexes harbouring the WSN PB2 subunit (W/W/W, H/H/W) and complexes containing the VN1203 PB2 protein (H/H/H, W/W/H). Collectively, these results showed that the VN1203 complex is active at a broad range of temperatures, that this property is dependent on the PB2 subunit, and that this phenotype can be transferred to the WSN 3P complex simply by exchanging the PB2 subunit.

### An avian PB2 subunit renders a mammalian WSN complex transcriptionally more active

**Fig. 4.**
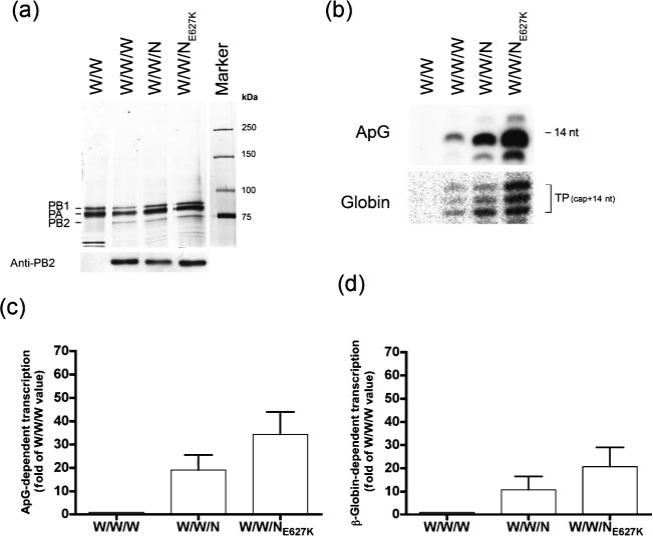
An avian PB2 subunit increases the activity of the WSN 3P complex at 30 °C. (a) Purified 3P complexes from the WSN (W/W/W) virus were prepared, along with complexes in which the PB2 subunit was omitted (W/W) or substituted by the PB2 from the avian Nanchang virus (W/W/N) or a mutated derivative (W/W/N_E627K_). The purified complexes were detected by silver staining and immunoblotting against PB2 (top and bottom panel, respectively). PB2-normalized complexes were used in functional assays (30 °C). (b) The complexes were analysed for cap-independent (ApG) or cap-dependent transcription (top and bottom panel, respectively). The transcription products in the ApG primed assay (14 nt long transcript) and in the globin-primed assay (triplet band; capped primer +14 nt) were detected by autoradiography. (c, d) Quantitative data for the ApG- and the cap-RNA-primed assays are shown. The data were normalized in terms of fold activity over the activity of the W/W/W complex. The results shown represent mean transcriptional activity from at least three independent assays. Bars, sem.

In light of the putative avian origin of H5N1 viruses, such as A/Viet/1203/04, we wondered whether an avian PB2 (H3N2) protein might have similar properties to the human H5N1 PB2 protein. To test this hypothesis, we cloned and expressed the PB2 subunit from the A/chicken/Nanchang/3-120/01 strain. We then purified 3P complexes (PA/PB1/PB2) from the WSN strain as well as complexes in which the PB2 subunit was substituted with an avian PB2 (W/W/N) or a mutated avian PB2 (W/W/N_E627K_). An E to K mutation in an avian PB2 has been shown to significantly enhance polymerase activity in mammalian cells, as measured in transcription–replication experiments (Labadie *et al.*, 2007; Massin *et al.*, 2001). The purified complexes were analysed by silver staining and by immunoblotting against PB2 (Fig. 4a top and bottom panel, respectively). All complexes were able to initiate transcription at 30 °C in a cap-independent and a cap-dependent fashion (Fig. 4b top and bottom panel, respectively). Complexes containing the avian PB2 subunit were found to have 20-fold greater activity than the WSN 3P complex in the ApG-primed and 10–20-fold greater activity in the globin-primed transcription assay. Interestingly, in both assays an E627K mutation in the avian PB2 resulted in a slight increase in activity.

### An avian PB2 confers broad temperature tolerance on the WSN 3P complex

**Fig. 5.**
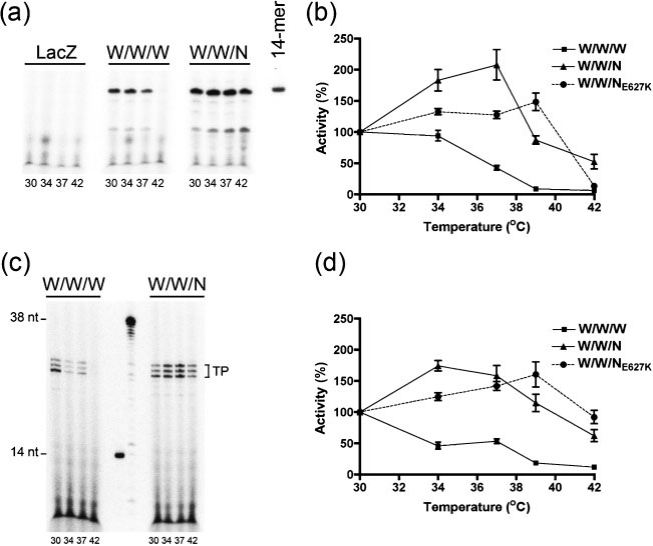
An avian PB2 subunit confers broad temperature tolerance on the WSN 3P complex independently of amino acid residue 627. Purified 3P complexes were prepared from the WSN virus (W/W/W), along with complexes in which the PB2 subunit was substituted by an avian PB2 (W/W/N) or a mutated derivative (W/W/N_E627K_); an extract prepared from LacZ-expressing cells (LacZ) was used as a negative control. Equivalent amounts of polymerase complex (as determined by polymerase activity at 30 °C) were tested for transcriptional activity at different temperatures (30, 34, 37, 39, 42 °C). Representative autoradiograms (a, c) and quantitative data (b, d) are shown for the ApG- (a, b) and the cap-RNA-primed (c, d) assays. The data were normalized in terms of percentage of the amount of fully extended product at 30 °C (as quantified by Phosphorimager analysis); the results shown represent mean transcriptional activity from at least four independent assays. Bars, sem.

The purified complexes (W/W/W, W/W/N) were evaluated for their activity over a range of temperatures by using an equivalent amount of functionally active complex as described above. In the ApG- and cap-primed transcription assay (Fig. 5a and c) the W/W/N complex had optimal activity at 30–39 °C (peaking at 37 °C and modestly declining at 42 °C). We then quantified the transcriptional activities of all three complexes (W/W/W, W/W/N, W/W/N_E627K_) in the ApG- and cap-primed transcription assay (Fig. 5b and d, respectively) and found, that an E627K mutation slightly shifted the temperature optimum from 37 °C to 39 °C.

Collectively, these results suggest that an avian PB2 subunit behaves similarly to the VN1203 (H5N1) PB2 subunit, and that it too enhances the activity of the WSN 3P complex at elevated temperatures.

### An avian PB2 confers enhanced functional stability on the WSN 3P complex

**Fig. 6.**
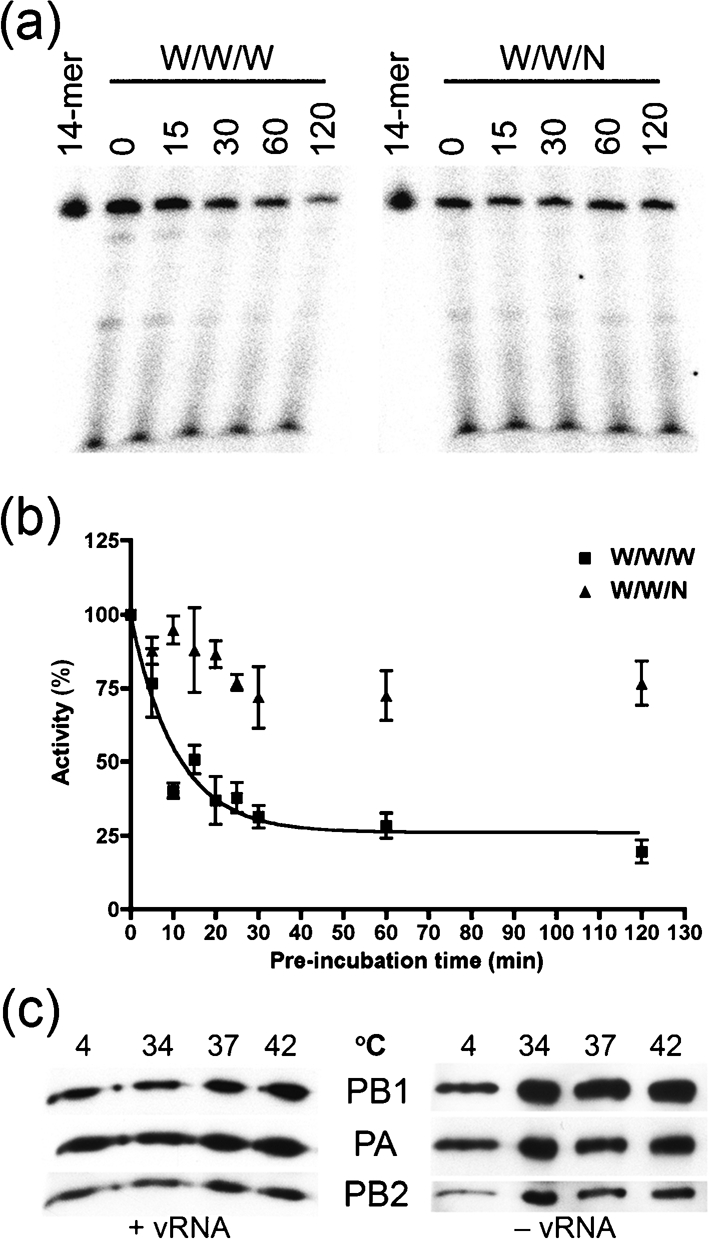
An avian PB2 subunit confers enhanced thermostability on the WSN 3P complex. Equivalent amounts of polymerase complex (as determined by polymerase activity at 30 °C) were pre-incubated at 30 °C in the absence of vRNA for different time periods. The complexes were then tested in an ApG-primed transcription assay (30 °C, 1 h). (a) A representative autoradiogram and (b) quantitative data from replicate experiments are shown. The data were normalized to the amount of fully extended product where no pre-incubation was performed (as quantified by Phosphorimager analysis). Mean transcriptional activities from at least four independent assays are shown. Bars, sem. A nonlinear curve for a single-phase exponential decay was fitted to the data using GraphPad Prism. The *r*^2^ value (goodness of fit) for the W/W/W curve was 0.81, and the half-life of the W/W/W complex was estimated at 7.3 min (with 95 % confidence intervals ranging from 5.3 to 11.8 min). The half-life of the W/W/N complex could not be determined, as it exceeded the 2 h of our analysis. (c) The purified WSN 3P complex was pre-incubated at the indicated temperatures in the presence (left panel) or absence of both 5′- and 3′-vRNA (right panel). The proteins were pulled-down with NiNTA Magnetic Agarose Beads and detected by immunoblotting with antibodies directed against PA (anti-His), PB1 and PB2.

To investigate the mechanistic basis for the increased thermal tolerance of complexes containing the avian PB2 subunit, we conducted a comparative analysis of the thermostability of the W/W/W and the W/W/N complexes. For this experiment, an equal amount of 3P complexes (as determined by functional activity at 30 °C) was preincubated at 30 °C. This occurred in the absence of any vRNA or nucleosides over different time periods (0–120 min). The transcriptional activity of each complex was then measured (30 °C, 1 h). It has been reported that the WSN 3P complex is heat labile in the absence of vRNA (Brownlee & Sharps, 2002), which was confirmed by our analysis. For the W/W/W complex we observed an exponential decay in transcriptional activity when pre-incubated at 30 °C (Fig. 6a, b); the half-life of the complex was estimated at 5–12 min at this temperature (Fig. 6b). However, the W/W/N complex was very stable over the entire time-period – with a loss of activity of only about 25 % over a 2 h period. These results demonstrate that the avian PB2 subunit confers greater stability on the WSN 3P complex and extends the functional half-life of the complex. While the functional activity of the W/W/W complex rapidly declined during incubation at 30 °C, this could not be attributed to simple physical dissociation of the complex, since it remained intact even in the absence of vRNA (Fig. 6c).

## DISCUSSION

Some H5N1 avian influenza viruses (Claas *et al.*, 1998; Li *et al.*, 2004) exhibit high pathogenicity in humans with pandemic potential (Gambotto *et al.*, 2008). This underscores the need to better understand the mechanisms that determine the ability of emerging influenza viruses to cross the species barrier into humans. This process is regulated in part by the viral haemagglutinin (HA) and host sialic acid receptors (Suzuki *et al.*, 2000). The viral polymerase complex is also recognized to play an important role in IAV host-cell specificity and pathogenicity (Gabriel *et al.*, 2005, 2007; Labadie *et al.*, 2007; Massin *et al.*, 2001; Salomon *et al.*, 2006), but the underlying basis for this remains poorly understood.

In this report, we show that the IAV 3P complex can be successfully purified from human lung epithelial cells using an adenoviral system. We analysed the functional activity of IAV polymerase complexes from the WSN (H1N1) and VN1203 (H5N1) strains, as well as chimeric complexes in which the PB2 subunits were exchanged. Our data show that the H5N1 polymerase complex has more robust thermotolerance than its WSN counterpart, which is active over a narrow temperature range (30–34 °C) with a more than 50 % reduced activity at 37 °C. This effect can be attributed to the PB2 subunit, since a simple exchange of this protein was associated with substantial changes in the temperature sensitivity of the polymerase activity. This effect does not depend on the presence of a glutamic acid residue at position 627, since the PB2 subunits from both the VN1203 and the WSN strain harbour a lysine at position 627 (K^627^). This suggests that additional determinants within PB2 contribute to virus host-range and replication efficiency as well (Gabriel *et al.*, 2005; Naffakh *et al.*, 2000; Shinya *et al.*, 2004).

We also generated complexes in which we substituted the PB2 subunit in the WSN 3P complex with an avian (H3N2) PB2 counterpart. Functional analysis of these complexes revealed that, like the H5N1 PB2 subunit, the avian PB2 subunit also conferred improved thermotolerance on the WSN 3P complex. Associated host-factors could possibly contribute to the functional properties of the purified 3P complex, although future experiments will be needed to address this question.

Our analyses also showed that the E^627^ residue in the avian PB2 was not required for the enhanced functional activity of the chimeric W/W/N. The E627K mutation in the avian PB2 led to only a slight increase in thermotolerance. Studies that have described the role of PB2 amino acid residue 627 as a determinant of cold sensitivity of avian influenza viruses were performed using transcription–replication experiments (Labadie *et al.*, 2007; Massin *et al.*, 2001) or by studying the replication of infectious, recombinant viruses (Hatta *et al.*, 2007; Salomon *et al.*, 2006). These assays effectively examine both RNA transcription and genome replication, since a defect in either process would lead to a reduction in measured overall replication. In contrast, our experiments examined only viral transcription. Since viral transcription and replication occur via distinct mechanisms, our findings suggest that PB2 residues other than amino acid 627 may regulate temperature sensitivity of viral transcription – and that mutations at these residues may contribute to virus-host adaptation and temperature sensitivity of overall virus replication, through effects on RNA transcription (Chen *et al.*, 2006; Finkelstein *et al.*, 2007). Further studies to address this hypothesis are ongoing.

At the mechanistic level, our results show that the thermotolerance-enhancing activity of the avian PB2 can be attributed to an increase in functional stability of the polymerase complex. Brownlee & Sharps (2002) have speculated that, in the absence of the vRNA promoter, the WSN polymerase complex is open and labile to heat inactivation. We showed that the half-life of the WSN complex in the absence of vRNA was roughly 10 min at 30 °C, whereas the functional half-life of the W/W/N complex exceeded 2 h. The short functional half-life of the WSN complex was not due to disintegration or disassembly of the tripartite polymerase protein complex, since the complex remained intact for at least 1 h.

Our findings support the earlier hypothesis (Brownlee & Sharps, 2002) that avian IAV RNA polymerases may have increased heat stability, due to the need of avian viruses to replicate at elevated temperatures present in birds. Brownlee also hypothesized that highly pathogenic human influenza viruses may possess polymerases with increased thermostability, relative to less pathogenic strains. Our results provide support for both of these predictions, and suggest the need to examine the thermostability of RNA polymerases from additional viruses.

In conclusion, the results reported in this paper define a new and previously unappreciated role for the influenza PB2 protein in determining the thermostability of the virus RNA polymerase. Future studies will determine the specific amino acid residues that contribute to this property, and will assess whether increased thermostability is associated with greater pathogenicity and/or host adaptation by emerging influenza viruses.

## Supplementary Material

[Supplementary Figures]
