# Supplementary material for: The human H5N1 influenza A virus polymerase complex is active in vitro over a broad range of temperatures, in contrast to the WSN complex, and this property can be attributed to the PB2 subunit
Source: J Gen Virol. 2008 Dec;89(Pt 12):2923–32. doi: 10.1099/vir.0.2008/006254-0 (PMC3067610; doi:10.1099/vir.0.2008/006254-0)
Supplement: [Supplementary Figures] [file supp_89_12_2923__index.html]

 The human H5N1 influenza A virus polymerase complex is active in vitro over a broad range of temperatures, in contrast to the WSN complex, and this property can be attributed to the PB2 subunit -- Bradel-Tretheway et al. 89 (12): 2923 Data Supplement - Supplementary Figures -- Journal of General Virology

## 

### The human H5N1 influenza A virus polymerase complex is active *in vitro* over a broad range of temperatures, in contrast to the WSN complex, and this property can be attributed to the PB2 subunit, by B. G. Bradel-Tretheway, Z. Kelley, S. Chakraborty-Sett, T. Takimoto, B. Kim and S. Dewhurst

*Journal of General Virology* vol. **89**, part 12, pp. 2923 - 2932

**Supplementary Fig. S1.** The presence of PA/PB1 heterodimers does not affect the activity of the purified WSN 3P complex  [PDF]  (47 KB)

  
  
