# Supplementary material for: The human H5N1 influenza A virus polymerase complex is active in vitro over a broad range of temperatures, in contrast to the WSN complex, and this property can be attributed to the PB2 subunit
Source: J Gen Virol. 2008 Dec;89(Pt 12):2923–32. doi: 10.1099/vir.0.2008/006254-0 (PMC3067610; doi:10.1099/vir.0.2008/006254-0)
Supplement: [Supplementary Figures] [file supp_89_12_2923__1.pdf]

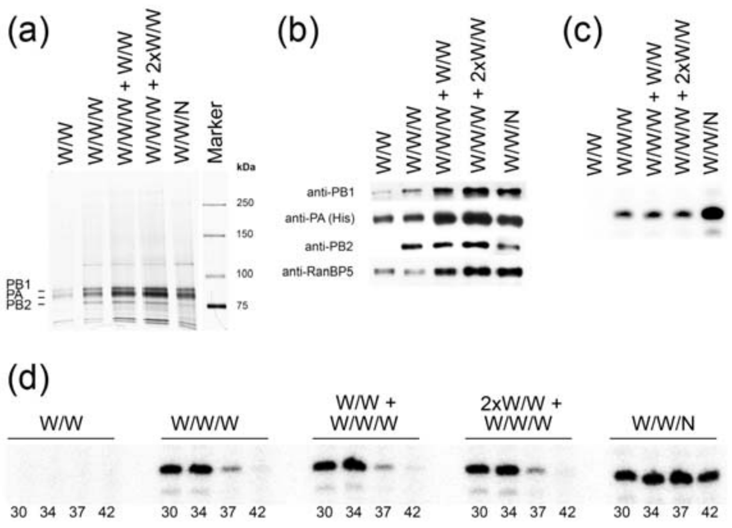

Supplementary Fig. S1. The presence of PA/PB1 heterodimers does not affect the activity of the purified WSN 3P complex. PA/PB1 heterodimer (W/W) was purified, as well as WSN 3P complex (W/W/W) and complex containing the PB2 protein from the avian H3N2 virus in place of the native WSN PB2 protein (W/W/N). In addition, increasing amounts of the purified PA/PB1 heterodimer (W/W) were added to the WSN 3P complex (W/W/W+W/W, W/W/W+2×W/W) to generate polymerase complexes containing an excess of the PA/PB1 heterodimer. (a) The purified PA/PB1 heterodimer (W/W) and the various trimeric polymerase complexes were separated by SDS-PAGE and then analysed by silver staining. The positions of the various proteins are indicated on the left side of the panel. Addition of increasing amounts of the PA/PB1 heterodimer resulted in the presence of an excess level of these proteins (compare the lanes marked W/W/W, versus those labelled W/W/W+W/W and W/W/W+2×W/W). (b) The purified PA/PB1 heterodimer (W/W) and the various trimeric polymerase complexes were separated by SDS-PAGE and then analysed by immunoblotting using antibodies directed against the indicated proteins (PB1, PA, PB2, RanBP5). The host cell factor RanBP5 is known to associate with the PA/PB1 heterodimer; its presence in the W/W/W complex therefore suggests the presence of low levels of residual PA/PB1 heterodimers within this material. As expected, the addition of excess exogenous PA/PB1 heterodimers to the W/W/W complex results in unchanged levels of PB2, but greatly increased levels of RanBP5 (compare the lanes marked W/W/W, versus those labelled W/W/W+W/W and W/W/W+2x W/W). (c) Equivalent amounts of the various trimeric polymerase complexes (as normalized in terms of PB2 protein content) were used in the ApG-primed transcription assay (30 °C). Shown is an autoradiogram of the results of the assay; the dark bands represent the fully extended transcription product. (d) Equivalent amounts of the various trimeric polymerase complexes (as normalized in terms of their functional polymerase activity at 30 °C) were evaluated for transcriptional activity at the indicated temperatures using the ApG-primed transcriptional assay. Shown is an autoradiogram of the results of the assay; the dark bands represent the fully extended transcription product. It can be readily appreciated that the presence of excess exogenous PA/PB1 heterodimers in the W/W/W complex had no effect on the temperature optimum of the polymerase activity of the trimeric protein complex (compare the lanes marked W/W/W, versus those labelled W/W/W+W/W and W/W/W+2×W/W).

Bradel-Tretheway, B. G., Kelley, Z, Chakraborty-Sett, S., Takimoto, T., Kim, B. and Dewhurst, S. (2008). The human H5N1 influenza A virus polymerase complex is active *in vitro* over a broad range of temperatures, in contrast to the WSN complex, and this property can be attributed to the PB2 subunit. *J Gen Virol* **89**, 2923–2932.
